# Dosimetric characterization of a novel UHDR megavoltage X-ray source for FLASH radiobiological experiments

**DOI:** 10.1038/s41598-023-50412-w

**Published:** 2024-01-08

**Authors:** Nolan Esplen, Luca Egoriti, Thomas Planche, Stephanie Rädel, Hui-Wen Koay, Brandon Humphries, Xi Ren, Nancy Ford, Cornelia Hoehr, Alexander Gottberg, Magdalena Bazalova-Carter

**Affiliations:** 1 Physics and Astronomy, University of Victoria, Victoria, V8P 5C2 Canada; 2 TRIUMF, Vancouver, V6T 2A3 Canada; 3 Chemistry, University of British Columbia, Vancouver, V6T 1Z1 Canada; 4 Physics and Astronomy, University of British Columbia, Vancouver, V6T 1Z1 Canada; 5 Oral Biological and Medical Sciences, University of British Columbia, Vancouver, V6T 1Z1 Canada

**Keywords:** Radiotherapy, Applied physics

## Abstract

A first irradiation platform capable of delivering 10 MV X-ray beams at ultra-high dose rates (UHDR) has been developed and characterized for FLASH radiobiological research at TRIUMF. Delivery of both UHDR (FLASH mode) and low dose-rate conventional (CONV mode) irradiations was demonstrated using a common source and experimental setup. Dose rates were calculated using film dosimetry and a non-intercepting beam monitoring device; mean values for a 100 μA pulse (peak) current were nominally 82.6 and 4.40 × 10^−2^ Gy/s for UHDR and CONV modes, respectively. The field size for which > 40 Gy/s could be achieved exceeded 1 cm down to a depth of 4.1 cm, suitable for total lung irradiations in mouse models. The calculated delivery metrics were used to inform subsequent pre-clinical treatments. Four groups of 6 healthy male C57Bl/6J mice were treated using thoracic irradiations to target doses of either 15 or 30 Gy using both FLASH and CONV modes. Administration of UHDR X-ray irradiation to healthy mouse models was demonstrated for the first time at the clinically-relevant beam energy of 10 MV.

## Introduction

The normal tissue sparing potential of ultra-high dose rate (UHDR) irradiation has been demonstrated using electron linear accelerators (e-linacs) in radio-biological studies spanning the past 40 years^1–3^. More recently, interest in the concept of UHDR radiation therapy (RT) has been re-kindled, driven in large part by the demonstration of differential effects between tumor and normal tissue - the so-called FLASH effect. Moreover, the advent of sophisticated new accelerators is facilitating delivery of various radiation beams with access to a wider variety of temporal configurations which may prove critical to the development of FLASH-RT techniques.

Unfortunately, the ability to deliver UHDR radiation for the purposes of FLASH-RT research is currently restricted by the poor availability of high intensity sources across all radiation modalities routinely employed for curative RT. At present, low-energy electrons ($E<10$ MeV) predominate the literature, but are of limited benefit to clinical translation owing to their limited depth of penetration^1,4,5^. UHDR compatible very-high energy electron (VHEE)^6,7^ and synchrotron ($\sim 100$ keV) X-ray sources^8,9^ have also drawn attention for FLASH research, but suffer from poor accessibility due to the limited number of research systems available worldwide, and current lack of compact alternatives. Conventional X-ray tube sources may address the need for accessibility but are limited in application due to the extremely low-energy beams produced^10–12^, though emerging compact X-ray technologies may help to alleviate this^13^. Protons, meanwhile, have attracted substantial interest in terms of FLASH development due to their favorable physics properties, but remain comparatively costly and thus rely upon established infrastructure to support their widespread adoption^14–16^.

In contrast to the above-stated particle modalities, megavoltage (MV) X-rays offer the ability to treat deep-seated tumors while requiring comparatively modest accelerator specifications. The development of MV X-rays therefore presents a high priority niche for future FLASH source research and development. Presently, systems capable of delivering UHDR (i.e. FLASH-compatible) X-rays at MV energies are at the developmental stage commercially^17^. Primary limiters to UHDR MV source development relate to engineering challenges associated with the design and fabrication of practical targets for bremsstrahlung conversion of sufficiently high-power electron driver beams. Globally, only two experimental MV X-ray FLASH beamlines presently exist - at the China Academy of Engineering Physics terahertz free electron laser (CTFEL, Chengdu, China) and TRIUMF (Vancouver, Canada) Advanced Rare Isotope Laboratory (ARIEL) facilities^3,18^. However, the source developed at ARIEL has proven to be the first to offer both UHDR and CONV MV irradiations on a common beamline, thereby affording a constancy in beam quality across these modalities. Consequently, the new FLASH Irradiation Research Station at TRIUMF (FIRST) stands as a unique research platform aimed at facilitating FLASH radiobiological experiments at heretofore unexplored energies (10 MV) and dose rates which can exceed 100 Gy/s^18^.

The aim of this work was to employ the FIRST 10 MV UHDR X-ray source in a pre-clinical study of FLASH effects in healthy lung of male C57Bl6 mice. To enable these experiments, it was necessary to first assess the system’s capabilities and characterize the beam under treatment conditions using established dosimetric techniques. For UHDR radiobiological research, accurate dosimetry is of critical importance in order to establish both reliable FLASH-RT delivery strategies and support reliable correlation between treatment methodologies and biological outcomes, especially in view of future translational research. Notably, careful consideration must be given to select detectors that are compatible with the temporal nature (i.e. pulsed or continuous) of the radiation source being employed^1,19–21^.

For the purposes of our small-animal irradiations at TRIUMF, radiochromic films (type EBT3) were selected for their demonstrated dose-rate independence over the range relevant to our UHDR X-ray source (i.e. $10^{-2}$ to $10^{2}$ Gy/s)^1,22^. This quality is especially important for the estimation of in-vivo doses in the absence of validated treatment planning systems or Monte Carlo models, and for experiments which consider a large range in dose rate (i.e. FLASH and CONV). The high spatial-resolution and energy independence of films at MeV beam energies^23,24^, for both photons and electrons, was also well-suited to the FIRST beam which boasts a broad energy spread, a small ($\approx 1\times 1\,\textrm{cm}^{2}$) field size and mixed photon/electron contribution down to $\simeq 2\,\textrm{cm}$ depth in water^18^.

To date, small-animal experiments for FLASH have been conducted for a range of animal models using electrons, protons, photons and carbon ions^8,14,25–27^. However, the mechanisms which drive the observed normal-tissue sparing (FLASH) effect remain to be elucidated. In order to better understand the limitations of FLASH, which includes identifying the pre-requisite beam delivery parameters and driving mechanisms, there exists a need to pursue in-vivo research under a much wider range of experimental conditions. For example, beam micro-structure and depth of anesthesia may have effects on the transient and persistent oxygenation status in tissue, respectively. How such factors might modulate observed FLASH outcomes are open questions which we might be better positioned to answer using emerging experimental systems. The ARIEL e-linac is particularly attractive for the proposed application owing to the range of applicable energies (300 keV to 30 MeV) and the ability to utilize highly flexible modes of current delivery, in contrast with the few other potentially FLASH-capable X-ray sources. The FIRST platform thus enables delivery of UHDR beams with flexible control over a number of source parameters of interest such as the (macro) pulse repetition frequency (PRF), peak current (i.e. instantaneous dose rate: $I_p\propto \dot{D}_p$), beam energy and average power ($P\propto \bar{\dot{D}}$).

The treatment of healthy mice throughout this project represents the culmination of an effort to enable FLASH-RT research using MV X-rays in Canada and demonstrate a world-first irradiation at 10 MV using a newly developed UHDR bremsstrahlung source. In this pre-requisite study, the capabilities of the FIRST system are discussed in detail using dosimetry results obtained through beam characterization and commissioning under both UHDR (FLASH mode) and conventional dose-rate (CONV mode) operation. Finally, the results of film dosimetry from the ongoing mouse radio-biological study will be summarized.

## Results

### Characterization of the FIRST irradiation platform

Radiochromic (EBT3) film measurements were conducted in solid water (SW) using parallel or perpendicular orientations relative to the beam direction, or perpendicularly within a water-filled Falcon tube (FT) at 1-cm depth which replicated the mouse irradiation setup. Together, the data allowed for robust field and depth dose-rate (DR) characterization which were pre-requisite to FLASH and CONV treatments of mice under identical conditions.

Dose rates ($\bar{\dot{D}}$) measured using sets of films in the SW phantoms demonstrated that UHDR X-rays with $\bar{\dot{D}}>80$ Gy/s were successfully delivered using the ARIEL e-linac at 10 MeV and a nominal beam power of 1 kW.

Mean dosimetric quantities for UHDR single-pulse irradiations in the FT were calculated from film dose and AC current transformer (ACCT) pulse-length measurements. The mean DR for a 100 ${\upmu }\textrm{A}$ (0.1 mA) peak beam current at 1 cm depth was calculated to be $82.6 \, \text {Gy/s}$ and the dose-per-charge ratio ($f_{FLASH}$) was ${0.75} \; \textrm{Gy}/\upmu \textrm{C}\,(\sigma =\pm 5.2\%)$. CONV DR (normal mode) irradiations were conducted under identical conditions as for the FLASH beam mode, but used the peak-current integration procedure outlined in the Methods. Fixed-length (300 s) irradiations with continuous beam delivery, a 100 Hz pulse repetition frequency, and 0.055% duty factor (DF) were conducted for both film-loaded FT or SW phantoms. The mean DR normalized to 100 $\upmu \textrm{A}$ (0.1 mA) peak beam current at 1 cm depth was calculated to be ${4.40\times 10^{-2}}$ Gy/s and the dose-per-charge ratio ($f_{CONV}$) was $0.74 \; \text{Gy}/\upmu {\textrm{C}}\,(\sigma =\pm 6.5\%)$.

**Figure 1:**
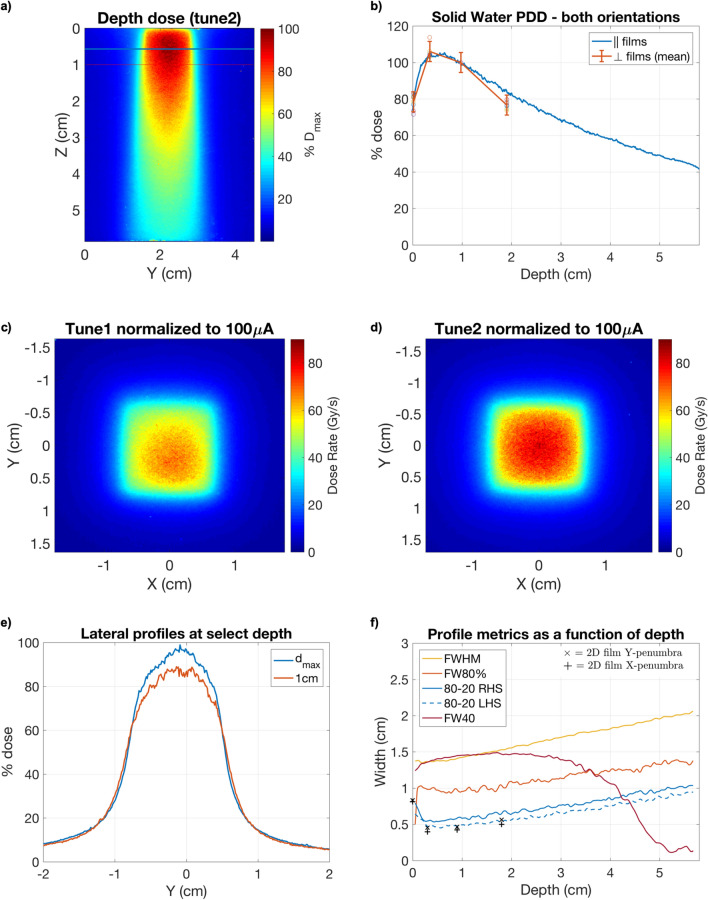
Parallel film percentage depth-dose map (**a**) and the corresponding PDD curve (**b**) normalized to 1 cm depth for the final beam tune. Results from 4 sample depths (at d = 0, 3, 9, 18 mm) using 8 sets of perpendicular films ($N=8$), adjusted for the approximate film thickness ($\approx {300} \; \upmu \textrm{m}$), are plotted as dots with error bar associated. Percentage errors are included for the averaged depth-dose result at each depth corresponding to the standard deviation in the mean. 2D dose-rate maps at 1-cm depth using both beam tunes, and normalized to an EMBD current of 100 $\upmu \textrm{A}$, are shown in (**c**) and (**d**). Lateral Y-profiles for $d_{max}$ (0.57 cm) and at the nominal treatment depth of 1 cm in solid water are shown in (**e**). The continuous depth-profile metrics for the Y-axis are summarized in (**f**) including the full-width at half-maximum (FWHM), full-width at $0.8D_{max}$ (FW80%), the 80–10% beam penumbra, and full-width at 40 Gy/s (FW40). The penumbra are calculated separately for the left (LHS) and right-hand sides (RHS). The mean penumbra values calculated from perpendicular film data are plotted for the Y and X dimensions denoted by the **x** and ${\textbf {+}}$ symbols, respectively.

Parallel SW films used for percentage depth-dose (PDD) and depth-profile measurements were analyzed and a representative dose map is shown in Fig. 1a. PDD results (Fig. 1b) have been compared against the discrete ($N=8$) depth-dose measurements taken from the perpendicular film data. Good agreement was found between the parallel and averaged perpendicular data, as evidenced by depth-doses agreeing within the bounds of the associated uncertainty down to a water-depth of 18 mm. At this point, a drop off in the perpendicular dose data appears due to the relative lack of backscatter in the 2-cm thick, perpendicular film phantom as compared to the parallel configuration for which the phantom thickness was 5.8 cm. Notable is the relatively low $d_{max}$ (0.57 cm) when compared with clinical 10 MV beams ($d_{max}\approx 2$ cm), which resulted from both the small field size, and reduced skin-sparing effect (higher superficial dose); the latter contribution is due to reduced self-attenuation and filtration of the beam in the converter target, as well as contaminant dose from low-energy electrons generated predominantly in the collimating structures. The minimal surface dose build-up was to be expected thanks to the large electron dose component, although it was more prominent as compared to previous Monte Carlo (MC) simulations, which has been attributed to the increased treatment source-to-surface distance (SSD) used in this study ($\simeq$ 8.5 cm)^18^.

Two-dimensional dose maps are shown for a perpendicular SW film sets in Fig. 1c,d, for two beam tunes, which qualitatively illustrate the relative degree of field symmetry. The first (tune1) and final (tune2) tunes were used throughout the 15 and 30 Gy mouse irradiation campaigns (see Mouse irradiation dosimetry), respectively. In between these campaigns, the beam was necessarily re-tuned and re-characterized following e-gun conditioning and facility shutdown. For the final beam tune (tune2), field symmetry was visibly improved in the dose distribution presented in Fig. 1d and was quantified by comparing the 80–10 penumbra widths for the parallel-film depth-dose profile (Fig. 1). The mean percentage difference across all depths was found to be ${9.6 \times 10^{-2}} \pm 2.2\%$.

Lateral (Y) dose profiles at $d_{max}$ and at 1 cm (dose prescription depth) are shown in Fig. 1e. While the consistency of the beam full-width at half-maximum (FWHM) and lateral penumbra is qualitatively apparent, there is a limited degree of flatness about the central-axis. The depth-dependence ($d\in [0,5.8]$ cm) of the beam Y-axis 80-20% penumbra and FWHM is presented in Fig. 1f. In addition, the full-width at 80% of dose-maximum (FW80) is shown, which encompasses the 0.5 mm$^{2}$ ($25\times 25$ pixels) region of interest (ROI) over which peak dose-rates were evaluated. A representative set of penumbra width measurements were plotted for a perpendicular film series and demonstrate agreement with the Y-axis parallel film data, with only subtle asymmetry implicated between X and Y field sides. The width of the beam region for which dose-rates exceeded 40 Gy/s (FW40) has also been presented for comparison against the FHWM and FW80. The depth at which the FW40 curve intersects FW80% ($d=3.6$ cm) delineates the maximum depth in water-equivalent media where the central 80% of the beam may be covered by $\bar{\dot{D}}>40$ Gy/s, wherein FW40 = FW80 = 1.25 cm. For comparison, the FWHM and FW40 curves intersect at a noticeably shallower depth of 1.35 cm, at which point FW40 measures 1.47 cm.

### Mouse irradiation dosimetry

The dosimetric results for the first in-vivo study of X-ray FLASH-RT at FIRST - including dose, DR, irradiation times along with the measured charge and experimental dose/charge conversion (*f*) factors - are summarized in Table 1 for the 15 Gy FLASH (FLASH-15) and CONV (CONV-15) cohorts. Table 2 correspondingly reports the results for the 30 Gy FLASH (FLASH-30) and CONV (CONV-30) cohorts.

**Table 1:** Dosimetric result summary of FLASH-15 and CONV-15 irradiated mouse cohorts ($N=6$ per group, $D_{1cm}=15$ Gy).

Mouse #	Beam mode	$D_{1cm}$ (Gy)	% difference^a^	Time (s)	Dose rate (Gy/s)	ACCT/EMBD actual ($\upmu \textrm{A}$)$^{\textrm{c}}$	Integrated charge ($\upmu$A s)$^{\textrm{c}}$	*f* (Gy/$\upmu$C)$^{\textrm{c}}$
1	FLASH	13.40	8.05	1.65 $\times 10^{-1}$	81.19	74.96	12.37	1.08
2$^{\textrm{b}}$	FLASH	14.76	1.30	1.54 $\times~10^{-1}$	95.83	76.00	11.70	1.26
3	FLASH	14.99	2.87	1.65 $\times~10^{-1}$	90.83	75.70	12.49	1.20
4	FLASH	14.49	0.58	1.71 $\times~10^{-1}$	84.71	71.90	12.29	1.18
5	FLASH	14.07	3.43	1.62 $\times~10^{-1}$	86.85	74.70	12.10	1.16
6	FLASH	15.72	7.89	1.71 $\times~10^{-1}$	91.93	76.90	13.15	1.20

Mean	FLASH	14.57	4.02	1.65 $\times~10^{-1}$1	88.56	75.03	12.35	1.18
$\pm \,\sigma \,(\%)$	FLASH	5.46	—	3.85	6.01	2.29	3.87	4.92
1	CONV	11.73	0.11	366	3.20 $\times~10^{-2}$	101.09	20.35	0.58
2	CONV	11.81	0.64	303	3.90 $\times~10^{-2}$	121.21	20.20	0.58
3	CONV	10.95	6.73	322	3.40 $\times~10^{-2}$	114.23	20.23	0.54
4	CONV	10.88	7.33	327	3.33 $\times~10^{-2}$	112.04	20.15	0.54
5	CONV	12.18	3.77	302	4.03$\times 10^{-2}$	121.79	20.23	0.60
6	CONV	12.89	9.77	360	3.58 $\times~10^{-2}$	102.42	20.28	0.64
mean	CONV	11.74	4.72	330.00	3.57 $\times~10^{-2}$	112.13	20.24	0.58
$\pm \,\sigma \,(\%)$	CONV	6.46	–	8.33	9.24	7.94	0.34	6.32

$^{\textrm{a}}$The % difference is calculated as $|D-\bar{D}|/max\{D,\bar{D}\}$.

$^{\textrm{b}}$Beam trip; dose delivered as two consecutive shots (82 ms and 72 ms resp.), and current is the time-weighted average current.

$^{\textrm{c}}$Current and charge is measured with the ACCT or on the EMBD (target) readback for FLASH and CONV irradiations, respectively.

*f* is then applicable to the corresponding measurement device.

**Table 2:** Dosimetric result summary of FLASH-30 and CONV-30 irradiated mouse cohorts ($N=6$ per group, $D_{1cm}=30$ Gy).

Mouse #	Beam mode	$D_{1cm}$ (Gy)	% difference^a^	Time (s)	Dose rate (Gy/s)	ACCT/EMBD actual ($\upmu$A) $^{\textrm{c}}$	Integrated charge ($\upmu$A s)$^{\textrm{c}}$	*f* (Gy/$\upmu$C)$^{\textrm{c}}$
1	FLASH	29.61	0.14	2.59 $\times~10^{-1}$	114.33	150.00	38.85	0.76
2	FLASH	27.24	8.12	3.36 $\times~10^{-1}$	81.08	113.00	37.97	0.72
3	FLASH	30.84	4.02	3.36 $\times~10^{-1}$	91.80	121.00	40.66	0.76
4$^{\textrm{b}}$	FLASH	29.84	0.63	3.33 $\times~10^{-1}$	89.50	122.42	40.81	0.73
5	FLASH	31.70	6.90	2.92 $\times~10^{-1}$	108.55	137.00	40.00	0.79
6	FLASH	28.68	3.29	2.69 $\times~10^{-1}$	106.60	146.00	39.27	0.73

Mean	FLASH	29.65	3.85	3.04 $\times~10^{-1}$	98.64	131.57	39.59	0.75
$\pm \,\sigma \,(\%)$	FLASH	5.31	—	11.65	13.19	11.36	2.79	3.69
1	CONV	32.89	0.10	630	5.22 $\times~10^{-2}$	116.88	40.50	0.81
2	CONV	32.35	1.54	598	5.41$\times 10^{-2}$	122.86	40.41	0.80
3	CONV	33.23	1.15	627	5.30 $\times~10^{-2}$	117.27	40.44	0.82
4	CONV	32.68	0.54	499	6.55$\times~10^{-2}$	147.75	40.55	0.81
5	CONV	32.72	0.40	555	5.90$\times 10^{-2}$	132.91	40.57	0.81
6	CONV	33.26	1.22	600	5.54 $\times 10^{-2}$	122.52	40.43	0.82
Mean	CONV	32.86	0.83	584.83	5.65 $\times~10^{-2}$	126.70	40.48	0.81
$\pm \,\sigma \,(\%)$	CONV	1.06	–	8.54	8.82	9.33	0.17	1.11

$^{\textrm{a}}$The % difference is calculated as $|D-\bar{D}|/max\{D,\bar{D}\}$.

$^{\textrm{b}}$Beam trip; dose delivered as two consecutive shots (97 ms and 236 ms resp.), and current is the time-weighted average current.

$^{\textrm{c}}$Current and charge is measured with the ACCT or on the EMBD (target) readback for FLASH and CONV irradiations, respectively.

*f* is then applicable to the corresponding measurement device.

For both the 15 and 30 Gy groups, measured DRs at 1-cm water-depth for the 10 MV beam were > 80 Gy/s and > 0.03 Gy/s for FLASH and CONV modes, respectively. This verifies the successful delivery of UHDR treatment, as well as sufficiently low-DR for CONV.

In the 15 Gy groups, the FLASH-15 cohort received, on average, 14.6 Gy ± 5.5%, while the CONV-15 cohort received a lower 11.7 Gy ± 6.5% dose. A spurious DR drop prior to CONV initially resulted in the lower dose delivered; this is evidenced in the substantial reduction in $f_{CONV}$ compared to the calibration data. This lower dose prescription was intentionally maintained across all subjects in order to allow consistent evaluation of toxicity effects within the CONV-15 cohort. Mean delivery times to 15 Gy were 163 ms for the FLASH pulses and 5.5 min for the CONV irradiations.

Similarly, among the 30 Gy groups, the FLASH-30 cohort received, on average, 29.7 Gy ± 5.3%, and the corresponding CONV-30 doses were 32.8 Gy ± 1.1%. Evidently, there was a reduction in dose variance for the CONV-30 cohort relative to either CONV-15 or either of the FLASH cohorts, which is ascribed to improved procedures following the experiences with the 15 Gy group. Moreover, a $\approx 10\%$ overdose relative to the 30 Gy prescription was observed contrasting with the underdosing in the CONV-15 group. This was presumably due to an under-estimation of the $f_{CONV}$ conversion factor, as evidenced by the value calculated during the mouse irradiations (Table 2).

## Discussion

This work concerns the first recorded demonstration of UHDR, sub-second irradiations performed using the FIRST experimental 10 MV X-ray beam at the TRIUMF ARIEL e-linac facility. Film dosimetry and real-time current measurements offered the high spatial and temporal resolution necessary to demonstrate the system’s capacity to deliver a UHDR 10 MV X-ray beam suitable for FLASH-RT research, and enabled consistent dosimetry methods for in-vivo experiments.

UHDR irradiations using the FLASH mode demonstrated that dose-rates in excess of 80–100 Gy/s could be obtained for the beam currents typically employed ($\sim {0.1 \; }\textrm{mA}$). By contrast, DR $<0.1$ Gy/s was achieved in CONV mode at 100 Hz using a low 0.055% DF. Based on subsequent mouse irradiations, it was demonstrated that doses could be delivered to within 10% of the prescription using the delivery strategy developed. In general, delivery of FLASH and CONV was predicated on careful pre-treatment dose measurements, whose uncertainties were dominated by variance in the electron source output and of the film dosimetry itself. In this work, beam characterization data were presented in detail for the final beam tune alongside the results from both mouse irradiation campaigns. For all absolute and relative dosimetry, EBT3 Gafchromic films were employed alongside real-time diagnostic systems for irradiation time and individual (macro) pulse measurements. Due to the high doses employed, a reduction in film dynamic range and dose sensitivity was expected for the prescribed doses of our mouse experiments. This prompted the use of the green color channel, typically used for doses >10 Gy^28–30^.

It was determined that the pulse shape and peak current stability were dependent upon the ambient conditions of the gun and radiofrequency (RF) power supply. There also existed day-to-day variation in the cathode heater voltage which was required to provide an optimal (square) pulse. Moreover, in continuous delivery mode the e-gun emission behaviour was dependent on the RF stability, itself tied to the RF power supply temperature. Active cooling with air-conditioning helped to mitigate temperature swings during the irradiation campaign and thus improve pulse-to-pulse reproducibility, as measured with the ACCT.

In general, doses were found to agree well between the perpendicular SW phantom results and FT measurements ($<3\%$ difference). These data sets were thus taken together when calculating the average dose-current conversion factors for the mouse treatments. FT measurements closely replicated the treatment conditions and provided a consistent means of evaluating the reference-dose ratio between reference and in-phantom film doses (i.e. $D_{ref}/D_{1cm}$), while SW data provided the two-dimensional field and depth-dose distribution. It should be noted that differences in backscatter conditions between the two SW phantom configurations likely explains the difference in doses at 2 cm depth although an air-gap effect may also be present^31^; therefore, only the perpendicular SW configuration was used for calibration and planning purposes so as to maintain consistency with the FT measurements and better reflect rodent morphology. While a full MC validation was not performed, it is interesting to note that the MC results for the dose rate (0.5 mm$^2$ ROI) at 1 cm depth in a 3D-printed mouse phantom ($\approx 86$ Gy/s) from the initial design study^18^ agreed to within 3.5% of the mean experimental DR (83 Gy/s) at 1 cm depth, calculated from the aggregate FT and SW measurements in this work. The experimental value in this case has been normalized to 1 kW average power and does not account for uncertainty in setup SSD, nominally set as 8.5 cm instead of 8 cm as used in the MC simulation. The effects of lung tissue inhomogeneity in the actual mouse was not corrected for, but the results of the initial design study point to a negligible perturbation at these MV beam energies. It is also interesting to compare the sharp depth-dose gradient and $d_{max}$ (0.57 cm) with existing UHDR electron sources. For example, the Kinetron 4.5 MeV electron beam used by Favaudon *et al.* (2014) exhibited a larger $d_{max}$ as compared to FIRST, between 1 and 1.3 cm, depending on the aperture size, but a comparable degree of dose buildup, or approximately $20\%$ (Fig. 1) over the domain of [0, $d_{max}$]^32^. Therefore, both FIRST and low-energy electron sources may offer reduced superficial dose buildup, as compared to a clinical 10 MV beam, but the MV X-rays employed at FIRST naturally afford a much more gradual dose fall-off beyond $d_{max}$. At 5 cm depth in water, the dose at FIRST is estimated to be $D({5} \; {\textrm{cm}})=0.5 D_{max}$, whereas negligible dose is recorded beyond the Kinetron beam range of approximately 2.5 cm^32^. Practically, this can at least double the tissue-depth for which $\bar{\dot{D}}>{40} \; \textrm{Gy}/\textrm{s}$ treatments might be realized at FIRST, at least when compared with the non-VHEE electron beams found within the FLASH literature^22,32,33^. Nevertheless, the presence of steep superficial depth-dose gradients leads to dose heterogeneity issues which currently limit the applications of these sources beyond preclinical work and must be resolved in view of future translational research involving MV X-ray UHDR sources.

All mouse irradiations were successfully conducted for both FLASH and CONV groups in each of the 15 and 30 Gy cohorts. One mouse in each of the two FLASH groups had to receive a split dose due to beam trips during single-pulse delivery. The consequences of the split dose delivery for FLASH irradiation is not known and while it obfuscates the assessment of outcomes for these mice, it also represents an opportunity to note any differences or lack-thereof. In terms of treatment reliability, the greatest precision in dose delivery (lower mean $\sigma$) was observed for the CONV-30 group. FLASH irradiations, on the other hand, were more accurate but featured a higher standard deviation in dose, which primarily reflected the intrinsic fluctuations in peak current and therefore charge-per-pulse. It is important to note that an accelerator shutdown period necessitated e-gun conditioning and beam tune revision following treatment of the 15 Gy cohort. Following the mid-campaign shutdown period, re-characterization of dose-charge (*f*) conversion factors and X-ray beam metrics was necessary due to changes in the ACCT calibration and requisite beam tune. The extra refinement and improvements to source stability were reflected in the results for the 30 Gy cohort as a whole (Table 2). The 9.5% average over-dose observed for the CONV-30 mice was attributed to an under-estimation of the CONV mode dose-per-charge factor. Interestingly, the measured ACCT-EMBD charge ratio ($C_{ACCT}/C_{EMBD}\approx$ 1.10) was consistent with the results of the 30 Gy group for which $f_{m30,CONV}/f_{m30,FLASH}=1.11$, indicating that there may have been some unaccounted for factor which affected energy transmission at the target. For example, the total charge collected on the converter body may remain constant while X-ray transmission through the collimator assembly decreases as a result of changes in beam transport. This might include a shift in beam position on target due to continuous energy drift, which had been observed following longer periods of irradiation. Changes in secondary electron capture by the collimating body may also contribute to changes in measured current for a given dose. Future work could investigate the effect that electron beam position has on dose rate, and estimating the dose uncertainty that might be ascribed to current instabilities.

The in-vivo dosimetry results were collected for each treatment, as the beam conditions were liable to change between each lockup, and thus between mice. Notably, the prescribed dose could be consistently delivered with an accuracy of better than 10%, but there remains a need for improved understanding of beam parameter control in order to mitigate sources of dose error. Areas of improvement include understanding the sensitivity of the FLASH pulse shape to the cathode heater voltage, which otherwise introduced a DR time-dependence on the scale of milliseconds. Meanwhile, CONV mode peak-current instabilities were observed under conditions of high heat for the klystron power-supply. For longer irradiations, changes in RF and gun stability or ambient conditions might conceivably change beam output during or between sequential irradiations. While the quality of the beam tune and energy drift could be assessed visually using beam position monitors and a viewscreen for verification, it was difficult to assess how transmission through the accelerating cavity might be changing in time. These sources of output variability for the e-linac stress the importance of re-characterizing the beam ahead of each biological experiment. Ideally, a cross-calibrated, DR independent real-time dosimeter, such as an organic scintillating detector, would provide users a means to identify and correct for perturbations in the X-ray beam output independently of the ACCT.

Some notable difficulties arose in the delivery of CONV irradiation for both dose cohorts. Firstly, in the 15 Gy mouse cohort doses was under CONV irradiations were 19% lower than the FLASH doses. This was attributed to an initial overestimation of the DR. Moreover, instead of correcting the total charge delivered, the decision was made to retain the sample size ($N=6$) by prioritizing the delivery of the same dose to all members within the group.

Owing to manual targeting of the mouse thorax, it is likely that the treated mice received variable degrees of conformity to the total lung. This was due firstly to the fact that targeting was established using mechanical localization of the mouse position by means of the FT rather than animal-specific immobilization. Therefore, while the field area had a well-defined location within the FT holder, the mice could be subject to a small but unaccounted-for positioning error. Furthermore, manual targeting focused on the diaphragm and not the location of the lung during expiration phase, which could not be determined in situ without imaging capabilities; together, these factors could introduce inter-mouse variability and organ coverage dependent on the respiration phase. Secondly, even for a well-localized subject, the limited beam flatness and symmetry would introduce some degree of dose non-uniformity throughout the treatment volume. This might result in regions exposed to sub-UHDR ($<40$) Gy/s conditions. Because the mouse lung volume changes rapidly under injectable anesthesia, this situation is further complicated during FLASH pulses whose lengths are shorter than the respiration period. In effect, sub-second treatment would ‘freeze’ each mouse’s lung motion at different points within the breathing cycle. Unfortunately, this cannot be compensated for without beam gating and represents an inherent limitation to FLASH studies concerned with ultra-fast irradiation of organs subject to physiological motion, such as the lung or heart. Fortunately, all mice were monitored throughout the irradiation procedure using an infrared camera and no unexpected movement was observed. The long time under anesthesia poses an interesting question regarding induction of the FLASH effect, namely that there may exist changes in oxygenation status of animals under anesthesia, potentially eliciting an acute state of hypoxia, and driven in part by the change in respiration rate and altered metabolic activity^34,35^.

While the analysis lies beyond the scope of this work, pre- and post-RT respiratory-gated micro-CT scans are facilitating dynamic non-invasive tracking of radiation side-effect evolution, namely pneumonitis and lung fibrosis. The monitoring period for all groups was 6 months and imaging follow-up was conducted at 2, 4, 6, 9 and 12 weeks. The resulting data is expected to provide insight into relative toxicity for each modality and dose group across various endpoints based on histological and micro-CT image evaluation. For example, lung and tidal volume evolution and changes in CT number which accompany physiological changes are being monitored to assess transient changes in side-effect progression between each dose and modality configuration^36^. All of the mice in the FLASH-15 and CONV-15 treatment groups survived to 24 weeks. One mouse in the FLASH-30, and 2 mice in the CONV-30 treatment groups reached humane endpoint and were euthanized early. The remaining 30 Gy mice survived to 18 weeks. All surviving mice within both 30 Gy exposure groups exhibited damage to the lung (4/4 CONV and 5/5 FLASH), with greater variation in the size of the damaged region in the FLASH mice (see Supplementary Fig. S1). Interestingly, damage to the lungs generally appeared to increase at the periphery of the right-lateral side corresponding to the beam entrance, extending inward towards the parenchyma for higher dose. This may implicate a spatial, possibly dose-related, dependence in local tissue damage that remains to be elucidated. A radiobiological follow-up study for the in-vivo irradiations described in this work is the subject of a forthcoming publication^37^.

At present, one of the primary limitations with the ARIEL FIRST platform is the sample throughput, bound by the infrastructure and accelerator hall access routine. Currently, only 1 sample/phantom pair ($N=2$) or a single mouse (to preserve bunker position use), can be irradiated every $\approx$ 45 min accounting for a full installation/lockup, delivery and access/retrieval. This limited also our feasible cohort size (statistical power) and demanded greater depth of anesthesia using injectables along with associated experimental risk.

The ability to reliably deliver treatments compatible with FLASH radiobiological experiments depends on reproducible beam delivery and reliable calibration. In this first campaign with the new FIRST user platform at ARIEL, good success was found employing a correction scheme, as exemplified by the relatively low dose variance amongst irradiated subjects in spite of changing beam conditions (see Table 1). Importantly, both CONV and UHDR irradiations were made possible at FIRST under the same treatment conditions which minimizes any response variance due to differences in beam quality or irradiation setup. Unfortunately, treatment reproducibility is still challenged by the unexpected transmission changes between modes, FLASH-pulse instability, noise in the e-gun current and energy drift. Together, these factors limited our ability to base delivery parameters on previously measured benchmarks and highlights the importance of implementing real-time dose measurement capabilities. In addition to introducing ACCT monitors after the accelerating cavity in the EMBD beamline, implementing active dosimetry and in-air beam imaging, such as through the use of scintillating screens or fiber-optic dosimeters, could provide substantial improvements to our understanding of the X-ray source and improve planning capabilities for future experiments.

## Conclusion

Research concerned with the normal-tissue sparing effects of ultra-high dose rate (FLASH) radiotherapy is of high importance to the oncological community, but few sources exist globally which are capable of achieving the requisite dose-rates. The ARIEL UHDR X-ray conversion target and FISRT irradiation platform for FLASH-RT research is the first of its kind in North America, and the first to reach the clinically-relevant energy of 10 MV worldwide. The modular experimental platform has been made suitable for dosimetric and biological irradiations and commissioned for both MV UHDR pulsed (FLASH mode) and low dose-rate (CONV mode) delivery, a first of its kind to do so.

In this first experiment on the new ARIEL FLASH system, UHDR X-ray beams were delivered with dose rates in excess of 100 Gy/s. Future stability improvements and a more flexible pulsed beam mode system could allow investigators to exploit the large beam parameter space (i.e. time structure) made available by the ARIEL e-linac. Mouse total-lung irradiations were carefully conducted using the best available dosimetry measurements and facilitated dose delivery to within 10 % of the prescription at 1-cm depth. Assessment of treatment outcomes will be subject to follow-up through future publication.

## Methods

The scope of this work concerns the characterization of the FIRST platform to inform requisite delivery parameters for in-vivo irradiation and dose verification. Dose measurements using radiochromic films in water phantoms (solid water or water-filled Falcon tube) allowed for field characterization at the relevant distance and depths in water. In-phantom film doses were related to doses measured using reference films located in-air between the X-ray source and sample stage. The resulting dose ratios were thus used to calculate doses delivered to mice at treatment depth based on the reference film measurements.

### Source Description

**Figure 2:**
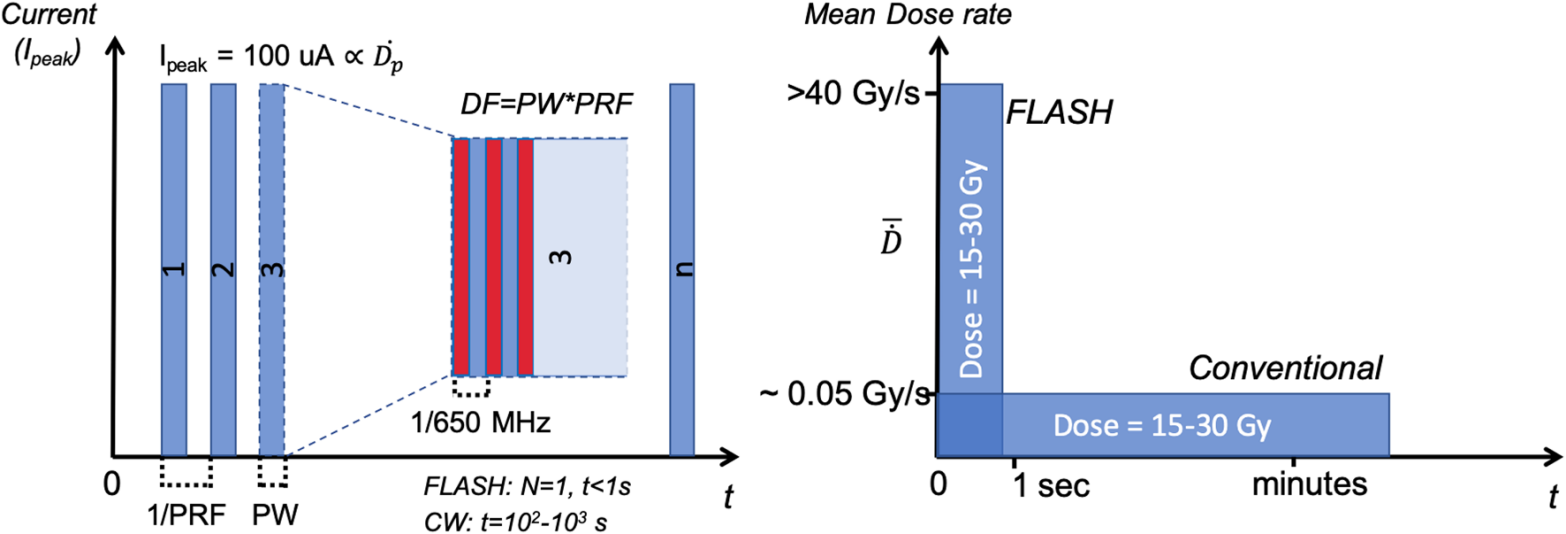
Beam time structure for conventional (CONV mode) and ultra-high dose rate (FLASH mode) delivery on FIRST.

The TRIUMF ARIEL superconducting electron linac is described in our previous work^18^. For the purposes of commissioning and subsequent radio-biological irradiations, only a subset of the available beam parameters were employed. Specifically, the electron beam energy was fixed at 10 MeV in order to maximize dose rates and target longevity^18^, while the beam (peak) current was nominally set between $95 \; {\text{and}} \; 105 \; \upmu \text{A}$ (± 5%). Current, and therefore dose, variation were unavoidable due to limitations with source stability^38^; the current variance increased at peak currents much lower than the e-gun design specification including those used for $P\le 1 \, \text{kW}$ operation in this experiment. To contend with this limitation, beam parameters were verified ahead of each irradiation and online adjustments made, as necessary, according to a standard procedure depending on the delivery mode. The e-linac has been commissioned to operate in either FLASH or CONV mode, which allowed for beam delivery using a single (macro) pulse or a defined PRF, respectively (see Table 3). To allow DR variation within either beam mode, the average beam current ($\bar{I}\propto P_{ave}$) was controlled by modulating a single parameter, the duty factor ($\textrm{DF}$) of the beam (Fig. 2). The DF represents the proportion of the repetition period (1/PRF) for which the beam is on, such that $\textrm{DF}/\textrm{PRF}=\textrm{PW}$, where PW is the pulse width. These source parameters enabled FLASH and CONV irradiation without perturbing the position, size and energy-spectrum of the beam during DR and mode selection.

**Table 3:** Summary of beam parameters available for FLASH and CONV irradiation on the ARIEL e-linac and those used for the dosimetry and in-vivo irradiation campaigns.

Parameter	Design values	Experimental values (nominal)	
		FLASH	CONV
Energy	8,10 MeV	10 MeV	
Average current	$\le 100\,\upmu \textrm{A} ({\pm } 10\%)$	$95{-}105\, {\upmu}$A	
Duty factor (DF)	0.05–100%	< 50%	0.055%
Frequency (PRF)	[1–100 Hz]	1 Hz	100 Hz
Pulse Width (PW)$^{\textrm{a}}$	PW=DF/PRF	< 500ms	5.5 ${ \upmu }$s
e-beam size ($2\sigma$)	2–10 mm	$\approx$ 5 mm	

$^{\textrm{a}}$DF= PW·PRF, where the PW for a FLASH macro-pulse is equivalent to a continuous beam delivery with total delivery time t= PW.

The approximate mean energy of the photon beam produced under either mode of operation was 1.76 ± 0.05% MeV, based on previous MC calculations^39^. The beam optics settings were established using beam envelope calculations^40^ from which the beam size (Gaussian 1$\sigma$) was found to be 2.27 mm in $\times$ and 2.79 mm in Y. The goal was to obtain a $2\sigma \approx 5 \; \textrm{mm}$ beam with zero dispersion at the location of the target and ensure that a maximally parallel and approximately Gaussian electron beam could be delivered. An energy spread of $<1\%$ could be maintained and phase shift adjustments to the accelerating section were applied as necessary to ensure the beam position and emittance remained consistent.

### Film calibration and dosimetry

Owing to their superior spatial resolution and established DR independence^1,22^, EBT3 Gafchromic® (Ashland Advanced Materials, Bridgewater, NJ) films were employed for the dose measurements used throughout the experimental campaign at TRIUMF. Prior to their first use for absolute dosimetry, the film batch was cross-calibrated against a reference 0.6cc ionization chamber (Farmer TN30010, PTW, Freiburg, Germany) and SuperMAX^TM^ electrometer (Standard Imaging, Middleton, USA) whose calibration is traceable to the standard laboratories of the National Research Council of Canada (NRC). The film calibration was handled according to supplier recommendations and TG-235 guidelines^30^ while the reference dose measurement was conducted according to the procedure set out by AAPM’s TG-51 protocol^41^. The films were located at a depth of 10 cm from the phantom surface at 100 cm SAD and the chamber reading was therefore scaled to the film depth by applying a previously measured tissue-maximum ratio conversion that was also verified at the time of irradiation.

In all, 18 calibration films were irradiated to equally-separated doses between 0 and 35 Gy using a 10 MV clinical photon beam delivered on a Varian TrueBeam STx (Varian, Palo Alto, USA). The feasibility of using EBT3 film beyond 20 Gy has been previously demonstrated^42,43^ though future work would benefit from the use of EBT-XD with enhanced dose sensitivity and accuracy out to $\approx 30-40$ Gy^42,44^. The energy independence of EBT3 films in the MV energy range was exploited to enable their use in our experimental beam despite the differences in beam quality as compared to the clinical 10 MV calibration beam^29,45^.

Irradiated films were scanned approximately 24 h after exposure on an EPSON^®^ 10000XL flatbed scanner (Epson America, Long Beach, CA) using the central-axis to minimize lateral response artifacts and a resolution of 200 dpi. A Matlab^®^ (Mathworks, Nattick, MA) script was written to process the scanned films using both red and green color channels, from which the absorbed dose was interpolated for each channel independently. Red channel responses were used for $\le$ 15Gy dose to exploit the improved dynamic range and reduced variance while for > 15Gy irradiations the green channel was instead used^29,30,46,47^. Our film uncertainty budget follows from the prescription of TG-235 and was ascribed to be $\pm ~5\%$, consistent with the literature comparing the red and green channels^47^.

### The FIRST irradiation platform and phantom setup

**Figure 3:**
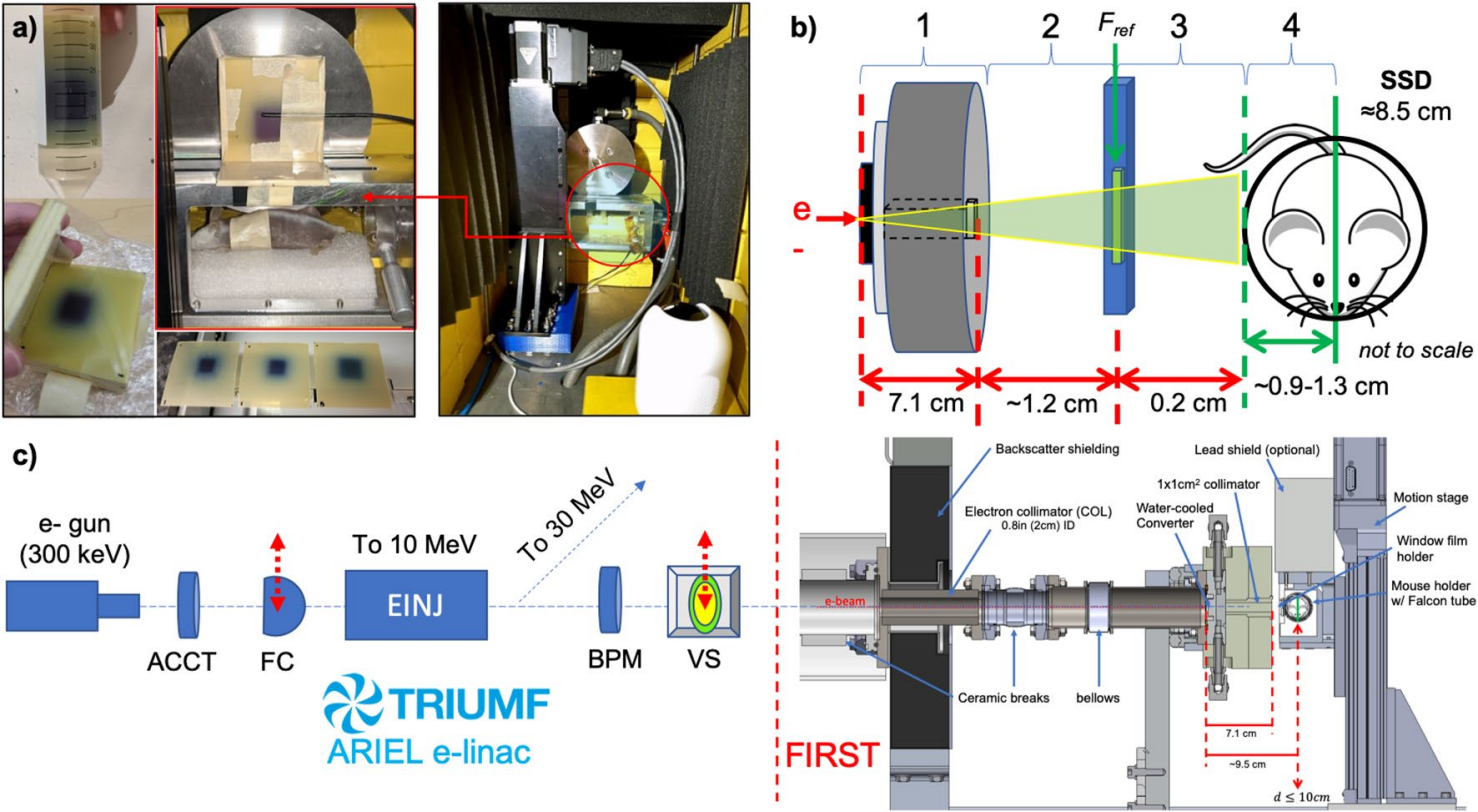
The new FIRST 10 MV X-ray FLASH platform has been installed downstream of the first accelerating section (EINJ) at the ARIEL electron linac. EBT3 film could be conducted in solid-water phantoms and a water-filled Falcon tube (FT) registered with the mouse treatment position (**a**). The relative position of the in-air reference film (F_ref_) and the irradiation subject, either film-loaded phantoms or mice, is schematically illustrated in (**b**); included are the distances for source-to-collimator (1), collimator-to-film (2), film-to-surface (3) and surface-to-axis (4). The SSD is represented by the sum of distances $1-3$ and the water-depth accounts for FT wall thickness. Dose-to-charge conversion factors for FLASH and CONV were calculated at 1-cm depth in the FT water-phantom. The relative location of beam diagnostics (**c**, left), including the AC current transformer (ACCT), Faraday cup (FC), beam position monitor (BPM) and viewscreen (VS) relative to the FIRST platform (c, right) has been outlined. Important components of FIRST are annotated in the cross-sectional view of the shielded beamline section at FIRST (**c**) .

The target and supporting infrastructure is the culmination of a multi-year collaborative effort to design and fabricate a robust, cost-effective UHDR photon source (i.e. $> 40$ Gy/s) with the goal of enabling MV X-ray FLASH radiotherapy research in Canada (Fig. 3).

Following fabrication and prototype offline testing, the UHDR target system was commissioned for both UHDR pulsed (FLASH mode) and low DR (CONV mode) delivery. A key advantage of the system was the ability to switch between modes and DR regimes by simply changing the DF, keeping all other beam properties constant.

Notable commissioning milestones for FIRST included installation of a new AC current transformer (ACCT), an updated beam-dump (EMBD) and electron collimator (COL), temperature (K-type thermocouple) read-backs, cooling-water flow-rate monitoring, and the establishment of a zero dispersion beam tune. Flow rates were set to $\sim$ 7 lpm and beam current on the target/dump assembly was consistently between 105 and 120 $\upmu {\text {A}}$, consistent with conservative beam power (heat) load limitations determined through the previous design work^18^.

Diagnostics upstream of the target included a fluorescent viewscreen and non-intercepting beam position monitors for online beam centering and size verification, Faraday Cups (FC) for steady-state beam current measurement, and a calibrated ACCT for pulsed beam current measurement. While FCs are simple integrating charge collection devices, the ACCT presented a critical addition for measurement of the ultra-fast current transients which the FCs could not detect owing to the limitations of their associated readout electronics. Fundamentally, the ACCT functioned as an in-air induction toroid which detected changes in the current passing through it’s center on a millisecond time scale by using a rapid (70–300 MHz) 1.25 GSa/s RTB2004 oscilloscope (Rhode &Schwarz, Munich, Germany). The relative position of these components within the linac structure is illustrated schematically in Fig. 3c.

#### Solid water and Falcon tube phantoms

EBT3 Gafchromic film measurements were conducted in both a solid water (SW) stack comprising $3\textrm{-mm}$ thick slabs (CIRS Inc., Norfolk, VA, USA) and a water-filled 50 mL Falcon tube (FT). For SW measurements, discrete depth-dose profiles were acquired with films perpendicular to the beam direction at various depths within a $(5.8 \times 5.8 \times 3)\,\textrm{cm}^{2}$ phantom composed of 3-mm thick slabs. In addition, continuous PDD curves were estimated using films aligned parallel to the beam direction, but were not used for treatment dose calculations^31^. In either case, the phantom was placed on the top of the mouse holder mounted on the linear motion stage and carefully aligned by means of taking ‘scout’ films and measuring the required position shifts *a priori*. The stepper-motor driven motion stage (Zaber, Vancouver, Canada) had 10-cm of travel and was capable of vertically positioning the phantoms to within an accuracy of 25 $\upmu \textrm{m}$; meanwhile, the phantom position was localized horizontally using fiducials located 1 cm from the front edge of the mouse holder, which corresponded to an SSD of $\sim ~8.5$ cm. This setup was used to align the SW 1-cm depth-dose film with the location of the FT central-axis, which would in turn corresponded to the mouse mid-sagittal plane and treatment depth for subsequent in vivo irradiations.

**Figure 4:**
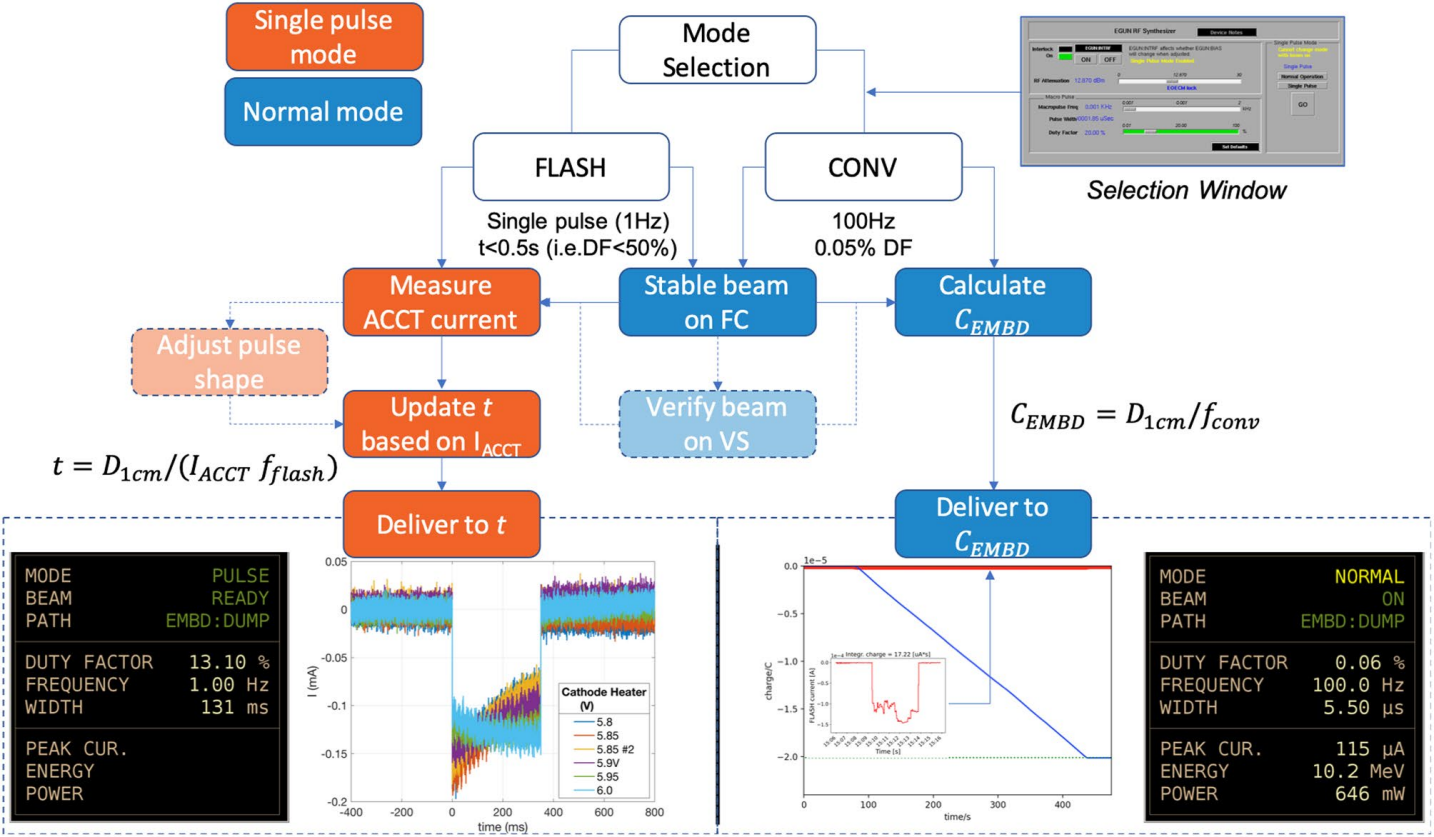
Beam delivery and monitoring workflow based on mode selection (CONV or FLASH) for radiobiological experiments on the ARIEL e-linac. The single-pulse (FLASH) or normal mode (CONV) operational elements are highlighted in orange or blue, respectively. Procedures with dotted lines denote aspects of beam delivery which are optionally followed based on operational needs. An inset (top-right) shows the controls screen for beam mode selection and beam temporal parameter adjustment. Examples of current monitoring outputs are shown in the bottom insets for pulsed FLASH (bottom-left; ACCT) and continuous CONV delivery (bottom-right; EMBD), including screenshots of the respective beam parameter summary screens.

To more closely reproduce the treatment setup of the mouse, and verify the doses measured within the SW stack, films were cut to fit within a water-filled polypropylene FT. Film submersion was limited to no longer than 1 h to avoid affecting results away from the taped film edges^48,49^. The results in this setup better reflected actual treatment conditions as the films could be positioned within the FT at the water depth representative of the mouse lung, $\sim 1$ cm assuming a nominal mouse diameter of 2 cm. The FT provided an additional 1 mm water-equivalent thickness due to the tube wall. In practice, this would have resulted in a reduction in electron skin dose for the mouse based on the steep dose-falloff at shallow depths observed in MC simulations^18^. Because doses could not be measured in-situ for mouse irradiations, the 1-cm depth-doses ($D_{1cm}$) measured in-phantom were correlated with dose delivered to (reference) films ($D_{win}$) located in-air, approximately $1-1.5\,\textrm{cm}$ upstream of the tube/mouse position (Fig. 4). The holder and films were thus rigidly fixed between the collimator at FT (i.e. SSD $\approx 8.5 \,\textrm{cm}$). A detailed schematic illustration of the setup is included in Fig. 3b where $\textrm{F}_{ref}$ represents the location of the reference films. Dose analysis ROIs comprised $25\times 25$ pixels about the geometric field center for all perpendicular (FT and SW) films.

During beam setup, just prior to irradiation, samples were removed from beam via the vertical motion stage to limit radiation dose to < 10 cGy prior to delivery and mitigate risk of accidental exposure. The sample holder was moved to a pre-defined treatment position once the beam was readied. In addition, the mouse holder was designed to accommodate a 25 kg lead brick on top of the motion stage adapter to further reduce the dose to samples in the bunker (lower) position.

### Irradiation procedure for UHDR and CONV delivery on the ARIEL e-linac

A summary of the procedure for delivering both 10 MV FLASH and CONV beams to the irradiation station are described herein. In all cases, the e-beam was provided by the thermionic e-gun at 300 keV and accelerated to 10 MeV through the accelerating cavity (Fig. 3). The peak current was set near to a nominal value of 100 $\upmu$A (0.1 mA) while delivery time or integrated charge corrections, required for FLASH and CONV modes respectively, were made online based on deviations from calibration values. Specifically, dose-to-charge (or current) conversion factors were established based on film doses (Fig. 3b), measured in-phantom, and the associated charge which was found by integrating the recovered ACCT response for FLASH or the total EMBD current signal during CONV. A schematic summary of the irradiation delivery workflow is shown in Fig. 4.

#### UHDR (FLASH) irradiation procedure

Beam was delivered at UHDR ($\bar{\dot{D}}\sim 10^2$ Gy/s) by ensuring the linac was set for ‘single pulse’ operation. In this mode, the PRF was 1 Hz and a variable DF modulated the total pulse length (irradiation time), up to a maximum of 1 s (Fig. 2. A practical limit for the pulse length was set at 500 ms to limit the risk of beam trips due to vacuum spikes or on the electron collimator, notably those resulting from spurious over-current on the target. The average power was limited to 1 kW based on thermomechanical and fatigue life limitations^18^ and corresponded to an average beam current of 0.1 mA at 10 MeV.

Prior to single-pulse delivery, steady-state (i.e. normal mode) beam was set-up on an upstream FC with which the peak current could be monitored. Once a stable beam had been achieved, the average current and shape for a single (macro) pulse could be acquired. To accomplish this, a single pulse was delivered with the FC inserted while the the ACCT oscilloscope recorded the pulse transient (see Fig. 4, bottom-left inset). The raw pulse data was immediately processed using a custom MATLAB (Mathworks, Nattick, MA) script to calculate the average (integrated) current ($I_{ACCT}$) and PW for the recovered signal. Since $I_{ACCT}$ was proportional to the mean pulse DR ($\bar{\dot{D}}$), the PW that was required to reach a prescribed dose - and thus the total FLASH irradiation time - was scaled in accordance with the measured ACCT current by using a pre-calibrated DR-to-$I_{ACCT}$ conversion factor (in Gy/µC):

1 $$\begin{aligned} f_{FLASH}=\frac{\bar{\dot{D}}}{I_{ACCT}} \end{aligned}$$

The irradiation time (t) required to deliver a prescribed dose to 1-cm depth ($D_{1cm}$) was calculated as:

2 $$\begin{aligned} t=D_{1cm}/(f_{FLASH}*I_{ACCT}) \end{aligned}$$

During preclinical treatments, the electron beam position on the target was verified by using the upstream viewscreen while the irradiation subject was kept in a retracted (shielded) position. Moreover, if the ACCT signal shape was highly non-uniform, adjustments to the cathode heater voltage were made to help stabilize and flatten output curves and thus minimize DR variations within the pulse. Once readied, the FLASH single-pulse mode was selected to deliver a single (macro) pulse.

#### CONV irradiation procedure

Beam was delivered at low dose-rates ($\bar{\dot{D}}\sim 10^{-2}$ Gy/s) by ensuring the linac was set for ‘normal’ mode operation, in which the DF - and thus the PW - as well as PRF were freely variable but were constrained by a maximum average beam power of 200 W, as determined by the following relation: $P_{ave}=(I_p\cdot E\cdot DF)<200\,{\text{W}}$, where $I_p$ is the peak current in mA, *E* is the beam energy in MeV and DF is the duty factor which represents the proportion of the time that the beam is on (see Fig. 2).

In contrast to FLASH irradiation, CONV delivery required much longer irradiation times at low DR, achieved using a low duty factor and fixed 100 Hz PRF. However, due to potential for beam current drift, it was insufficient to calculate *a priori* the treatment time based on measured current alone. Instead, a procedure was developed to deliver a prescribed dose based on the total integrated charge measured on the target/beam dump (EMBD). A dose-to-charge conversion factor was first calculated by delivering beam for a set amount of time and taking the mean ratio between film dose and the corresponding EMBD integrated charge according to the following formulation:

3 $$\begin{aligned} f_{CONV}=D_{1cm}/C_{EMBD} \end{aligned}$$

where $C_{EMBD}$ is the total integrated charge at the EMBD (i.e. on target). Thereafter, the charge (*C*) required to reach the desired dose to 1-cm depth in water could be determined from:

4 $$\begin{aligned} C_{EMBD}=D_{1cm}/f_{CONV} \end{aligned}$$

Figure 4 (bottom-right) shows an example of the (peak) current signal monitoring (red line and inset) and the corresponding integrated charge (blue line) which dictates the prescribed value where delivery should be terminated. The DF was nominally set to 0.05% for all irradiations which corresponded to a DR of approximately 0.05 Gy/s (see Results).

For phantom and animal irradiations, the current on the upstream FC was measured to verify the peak current, as for FLASH mode. Thereafter, sample irradiation would proceed as the beam current was integrated in real-time on the target read-back (EMBD).

### First UHDR and comparative CONV mouse irradiations at 10 MV

In a first small-animal study investigating X-ray FLASH at TRIUMF, four mouse cohorts each comprising six healthy male C57Bl/6J mice (Jackson Laboratory, Sacramento, CA, United States) were prescribed with single-fraction 15 Gy or 30 Gy total-lung (thoracic) irradiations using either FLASH ($>40$ Gy/s) or CONV ($<0.05$ Gy/s) beam modes and a $1\times 1\,\textrm{cm}^2$ field size. In all, 12 mice were irradiated for each modality with $\mathrm {N=6}$ per dose group, namely the 15 or 30 Gy FLASH (FLASH-15/30) or 15 and 30 Gy CONV (CONV-15/30). A control (sham irradiated) group ($\mathrm {N=6}$) was included and received only the injected anesthesia comprising Alfaxalone (40-60 mg/kg), Dexmedetomidine (0.15 mg/kg), and Butorphanol (2 mg/kg), delivered by subcutaneous injection.

All mice were aged 10-12 weeks at the time of treatment and were housed and monitored at the Centre for High-Throughput Phenogenomics (University of British Columbia, Vancouver, CA) where baseline (pre-IR) and follow-up micro computed tomography (CT) scans were conducted (at 2, 4, 6, 9 and 12 weeks post-IR)^37^. With the exception of the control group, treated mice were anesthetized on-site by a trained veterinary technician prior to transport to the e-linac bunker for installation on the FIRST platform. In all cases, mice were localized within a perforated 50 mL FT which was placed horizontally within the beam path. The end of the customized FT was cut off for access and replaced with a porous 3D-printed cap to ensure adequate airflow to the mouse while restrained and throughout irradiation. Mice were kept in a shielded position (by retracting the motion stage) until beam was ready for delivery; this procedure mitigated the risk of accidental X-ray exposure and allowed for safe low-power tuning with beam on target, verified through previous phantom measurements under identical setup conditions. An infrared camera enabled the health status of the mouse throughout treatment to be monitored, namely for consistent respiration and signs of anesthesia emergence, including tail movement and respiratory changes evidenced by the diaphragm.

The doses to mouse lung following irradiation were calculated using in-air (reference) film doses that were calibrated against depth-dose measurements acquired under identical setup conditions. One reference film was installed for each mouse irradiation in fixed position using a 3D-printed holder at a distance of 0.9-1.3 cm from the mouse central axis depending on the approximated mouse diameters and placement. A schematic/CAD illustration is provided in Fig. 3b. FLASH irradiations were delivered as single $<{0.5}\; {\textrm{s}}$ pulses while CONV was delivered using continuous (100 Hz) beam and a 0.055% duty factor. The summary of dosimetric results for the small-animal study as well as the average delivery parameters - including dose, DR, delivery time and charge measurements - is presented in the results section.

Prior to animal irradiations, ethics approval was obtained through the University of British Columbia (UBC) Animal Care Committee, in compliance with the Canadian Council on Animal Care (CCAC) assessment, for the experimental protocol (#A21-0060) under which all ARRIVE compliant animal procedures were conducted.

### Supplementary Information

Supplementary Information.

## Data Availability

Data is available from the corresponding author upon reasonable request.
