# Supplementary material for: Dosimetric characterization of a novel UHDR megavoltage X-ray source for FLASH radiobiological experiments
Source: Sci Rep. 2024 Jan 8;14:822. doi: 10.1038/s41598-023-50412-w (PMC10774358; doi:10.1038/s41598-023-50412-w)
Supplement: Supplementary file 1 — Supplementary Information. [file 41598_2023_50412_MOESM1_ESM.pdf]

Dosimetric characterization of a novel UHDR megavoltage x-ray source for FLASH radiobiological experiments: Supplementary Materials

Supplementary Results

Preliminary lung histology

All of the mice in the FLASH-15 and CONV-15 treatment groups survived to 24 weeks. One mouse in the FLASH-30, and 2 mice in the CONV-30 treatment groups reached humane endpoint and were euthanized early, with no histology available for these mice. The remaining 30 Gy mice survived to 18 weeks[1]. Hematoxylin and eosin (H&E) stained lung tissues were qualitatively analysed for varying indications of pneumonitis and fibrogenesis in order to assess the existence of modality dependent differences in radiation-induced lung toxicity. Figure 1 shows the H&E stained lung tissue from one mouse per treatment group at 5x magnification. These slides were selected for their pathology, having been identified as containing the most damaged tissue sections; therefore, the images are not representative of the entire lung or overall response, but illustrative of the relevant pathological endpoints. Images of the control mice all showed well-defined alveolar airspaces, with no disruptions. The mice that were exposed to 15 Gy had very similar appearance to controls. A few mice showed localized damage to the airspaces, with these damaged regions being quite small. Some of the damaged areas exhibited a disturbance to the shape of the lung as shown in 1a, and fibrotic tissue replacing the airspace in Figure 1b. Very small, localized regions of tissue damage were observed in 1/6 mice for the 15 Gy CONV RT group and 3/6 mice in the 15 Gy FLASH RT group. However, this assessment concerns a coarse sampling of the front, mid-line and back sections, whereby some pathologies may be missed, and does not account for the extent of damage when counting affected mice nor the differences in delivered dose between the modalities. The mice exposed to 30 Gy exhibited damage over larger regions of the lung, and had more fibrotic tissue replacing the airspaces (i.e. less airspace), as shown in Figure 1d,e. All of the surviving mice in both 30 Gy exposure groups exhibited damage to the lung (4/4 CONV and 5/5 FLASH), with greater variation in the size of the damaged region in the FLASH mice. Interestingly, damage to the lungs generally appeared to increase at the periphery of the right-lateral side corresponding to the beam entrance, extending inward towards the parenchyma for higher dose; this may implicate a spatial, possibly dose, dependence in local tissue damage that remains to be elucidated. Quantitative analysis of survival data and transmission imaging-derived metrics will be the subject of a forthcoming publication[1].

It should be noted that the histological samples showcase our endpoints within the most damaged lung tissue sections, emphasizing the pertinent pathology, but are not representative of the entire lung; therefore, an assessment of the relative biological effects that may be evidenced within the data has not been made herein.

Supplementary Methods

Histological follow up

Mice were euthanized by intraperitoneal injection of ketamine (300 mg/kg) and xylazine (10 mg/kg). The control mice, 15 Gy FLASH and 15 Gy CONV groups were euthanized at 24 weeks post-irradiation, and the 30 Gy FLASH and 30 Gy CONV groups were euthanized at 18 weeks post-irradiation. The lungs were excised and inflation-fixed by instillation of 10% formalin at 25 cm H2O to ensure the alveolar structures were properly fixed. The lungs were tied with suture at the trachea, and submerged in 10% formalin for at least 24 h for complete fixation. The lungs were paraffin embedded and sectioned coronally into 4μm thick slices, stained with hematoxylin and eosin (H&E) and mounted onto glass slides. Three or four slides, each containing 2 sections of the lung, were prepared per mouse at different depths within the lungs to ensure adequate sampling of the tissue. The slides were imaged on a Zeiss Axioplan microscope (Zeiss, Oberkochen, Baden-Württemberg, Germany) at 2.5x, 5x and 10x magnification. Lung fibrosis and pneumonitis were selected as endpoints for the histological determination and comparison of radiation-induced lung toxicity following CONV and FLASH treatments.

References

1. Ford, N. L. *et al.* Respiratory-gated micro-computed tomography imaging to measure radiation-induced lung injuries in mice following flash and conventional radiation therapy. *Int. J. Radiat. Oncol. Biol. Phys.* **(Submitted)** (2023).

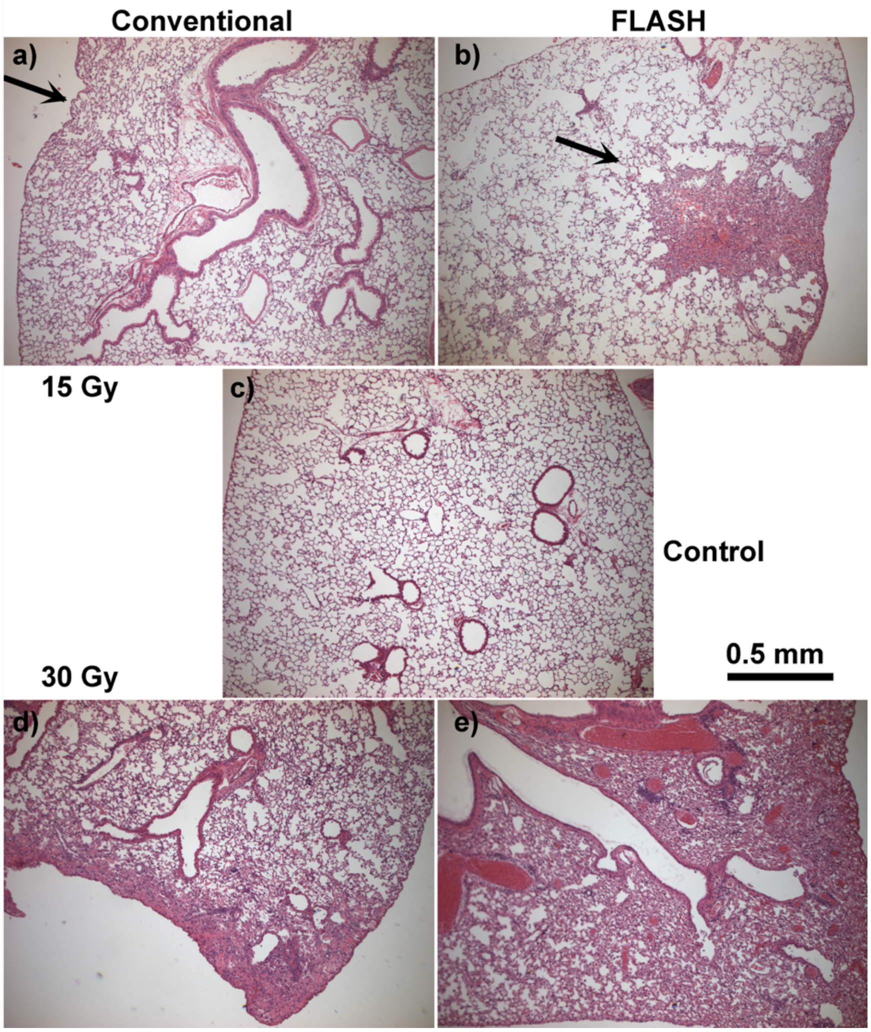

Figure S 1. H&E stained slides from one mouse per treatment group. (a) 15 Gy CONV RT, (b) 15 Gy FLASH RT, (c) unexposed control, (d) 30 Gy CONV RT, and (e) 30 Gy FLASH RT. The arrow in the CONV panel (a), identifies a region where the alveolar walls are thicker, potentially responsible for the local lung distortion. Meanwhile, the arrow in the FLASH panel (b) shows a localized region of damage, with the air spaces disrupted by the presence of fibrotic tissue.
